# Outcomes of damage control surgery in perforated sigmoid diverticulitis: a comparison before and after implementing a new treatment algorithm

**DOI:** 10.1007/s13304-026-02572-3

**Published:** 2026-02-27

**Authors:** Marie Burgard, François Pugin, Michel Adamina, Floryn Cherbanyk

**Affiliations:** 1 Department of Surgery, HFR Fribourg - Cantonal Hospital, Chemin des Pensionnats 2/6, 1752 Fribourg, Switzerland; 2 Faculty of Science and Medicine, University of Fribourg, 1700 Fribourg, Switzerland

**Keywords:** Diverticulitis, Damage-control surgery, Hartmann’s procedure, Emergency surgery

## Abstract

**Background:**

Non-restorative resection (NRR) is the most common procedure for perforated diverticulitis, but frequently leads to permanent stoma and morbidity related to stoma reversal. For selected patients, primary anastomosis (PRA) is another option. Damage control surgery (DCS) may be an alternative, potentially offering a greater likelihood of stoma-free discharge. We compared DCS to conventional surgical treatment in terms of stoma-free discharge and postoperative complications.

**Methods:**

This single center trial compared patients treated before and after the introduction of a new treatment algorithm based on DCS. The prospective study group underwent surgery from 2022 to 2024, following implementation of a DCS-based treatment algorithm. The retrospective control group underwent operation from 2020 to 2022 (pre-algorithm). The primary outcome was stoma presence at 12 months. Secondary outcomes included stoma at discharge, complications, length of hospitalization, costs, and complications after stoma reversal.

**Results:**

In the control group, 29/30 patients (97%) underwent NRR. In the study group, 22/26 (85%) underwent DCS, among these 4/22 (18%) required conversion to NRR, and 18/22 (82%) achieved anastomosis during the second-look. Demographics, length of hospitalization, post-operative complications, and complications after stoma reversal were similar. The control group exhibited higher rates of stoma at 12 months (37% vs. 8%, *P* = 0.003). Multivariate analysis revealed that treatment before the algorithm was independently associated with increased stoma presence at 12 months (Hazard ratio 10.65, *P* = 0.013).

**Conclusions:**

DCS appears to be a safe and effective strategy, yielding significantly lower rates of stoma at 12 months compared to conventional treatment for perforated diverticulitis.

**Graphical abstract:**

**Figure:**
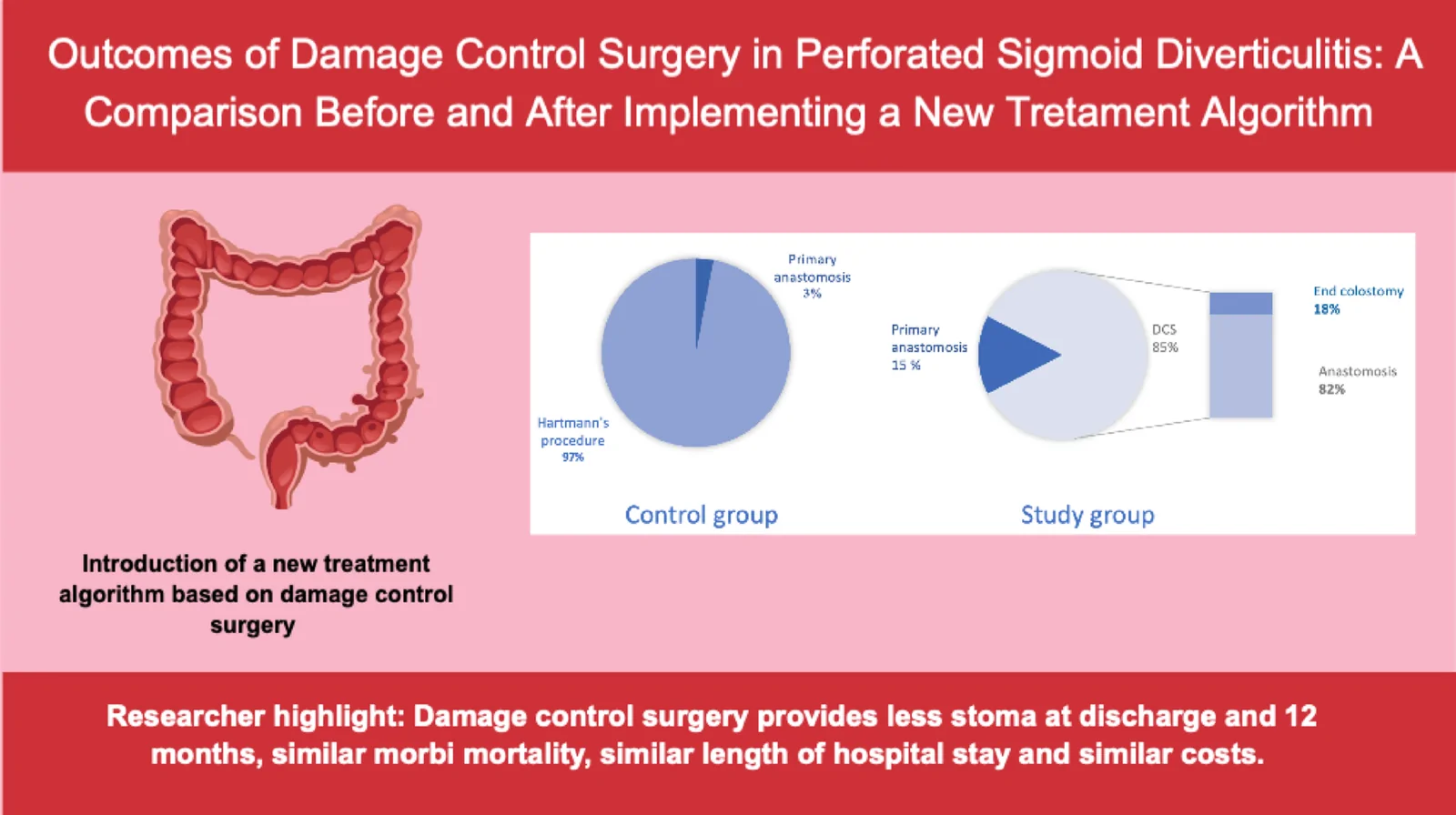

## Background

Perforated sigmoid diverticulitis with peritonitis is a potentially life-threatening condition. Current guidelines recommend emergency surgery for hemodynamically unstable or septic patients with complicated acute diverticulitis [1–4]. Over the past decade, numerous studies have explored the surgical approach options. Laparoscopic lavage is a viable procedure for selected patients with purulent peritonitis, while resection techniques should be used for patients with fecal peritonitis [1–5]. Debate continues over whether the optimal approach is non-restorative resection (NRR) or resection with primary anastomosis (PRA). While both procedures exhibit similar complication rates after the initial surgery [6, 7], NRR is associated with high colostomy non-reversal rates and significant reversal-related morbidity [6, 8, 9]. Indeed, about half of patients who undergo NRR do not have their stoma reversed [10, 11]. European and American guidelines suggest that resection with primary anastomosis may be performed in hemodynamically stable immunocompetent patients with perforated diverticulitis [1–4]. Nevertheless, the risk of life-threatening anastomotic leakage necessitates careful clinical consideration of patient age and comorbidities [3]. This decision-making process is further complicated by the fact that interventions for perforated diverticulitis often occur at night, and are performed by junior and non-colorectal surgeons. According to an American nationwide analysis published in 2019, 90% of surgeons still perform Hartmann’s procedure (NRR) in emergency settings [12]. 

More recently, damage control surgery (DCS) has attracted increasing interest for managing acute complicated diverticulitis [13–15]. It involves a rapid first-look laparotomy for source control and patient stabilization followed by a planned second-look laparotomy for definitive treatment. DCS has recently shown promise in non-trauma patients with intra-abdominal sepsis (IAS) [16] and perforated diverticulitis [13, 17, 18]. Studies of DCS treatment among patients with IAS have demonstrated low mortality rates and low stoma rates at discharge [10, 13, 16–18]. Therefore, DCS appears to be a viable alternative to NRR for critically ill patients with perforated diverticulitis. Although the World Society of Emergency Surgery Guidelines recommend DCS for unstable patients with diffuse peritonitis [3], European guidelines consider it to be a possible, but not yet established, technique and the literature is still scarce.

In this study, we aimed to compare a novel treatment algorithm for perforated sigmoid diverticulitis (based on PRA or DCS) to the conventional strategies (PRA or NRR), in terms of stoma-free discharge, early and late complications, costs, and stoma reversal.

## Methods

The study is reported according to The Strengthening the Reporting of Observational Studies in Epidemiology (STROBE) statement.

### Study design and patient inclusion

**Fig. 1:**
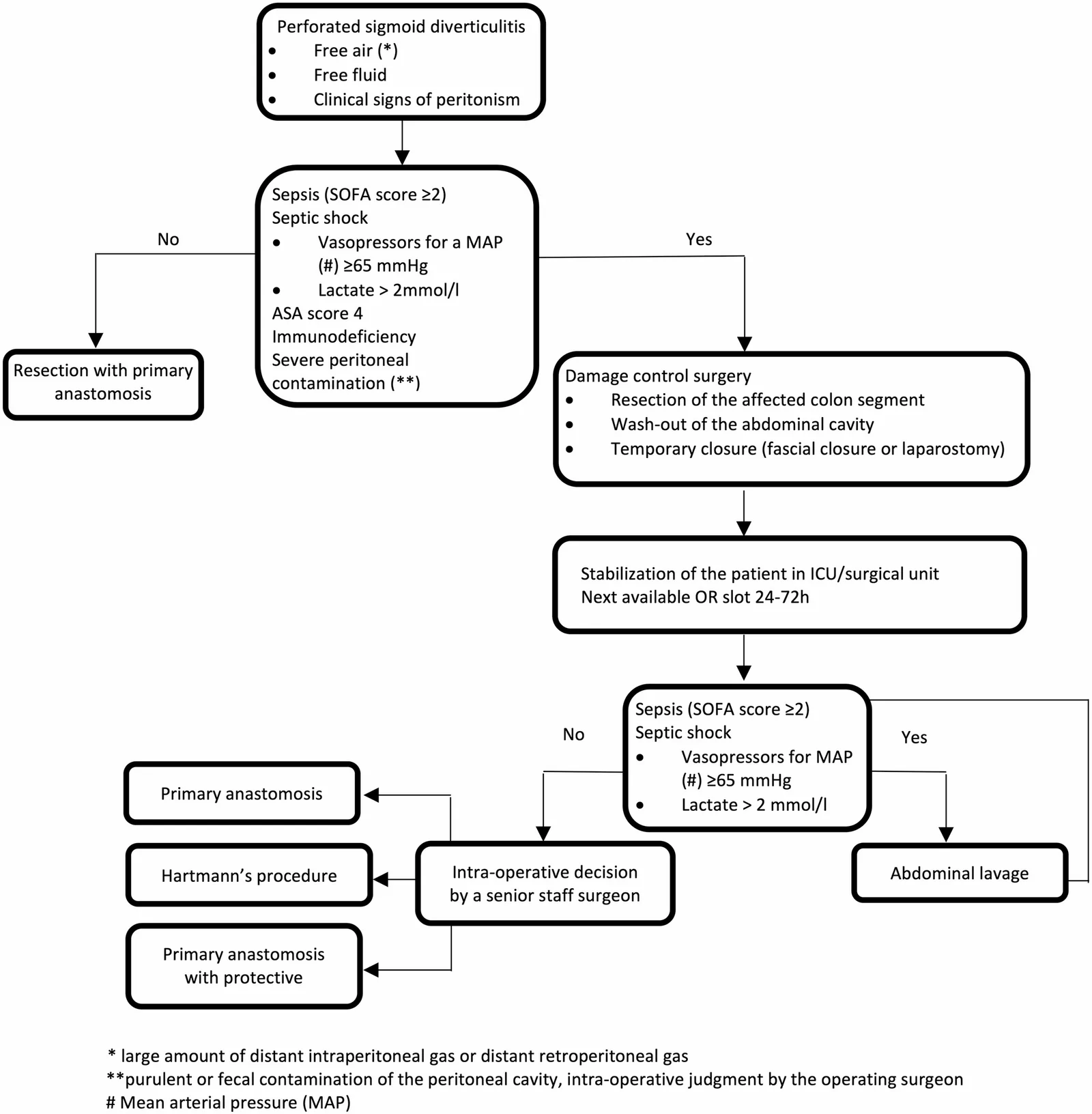
Novel two-staged damage control treatment algorithm. sofa = sequential organ failure assessment, MAP= mean arterial pressure, ASA = American Society of Anesthesiologists, ICU= intensive care unit, OR= operating room,

**Fig. 2:**
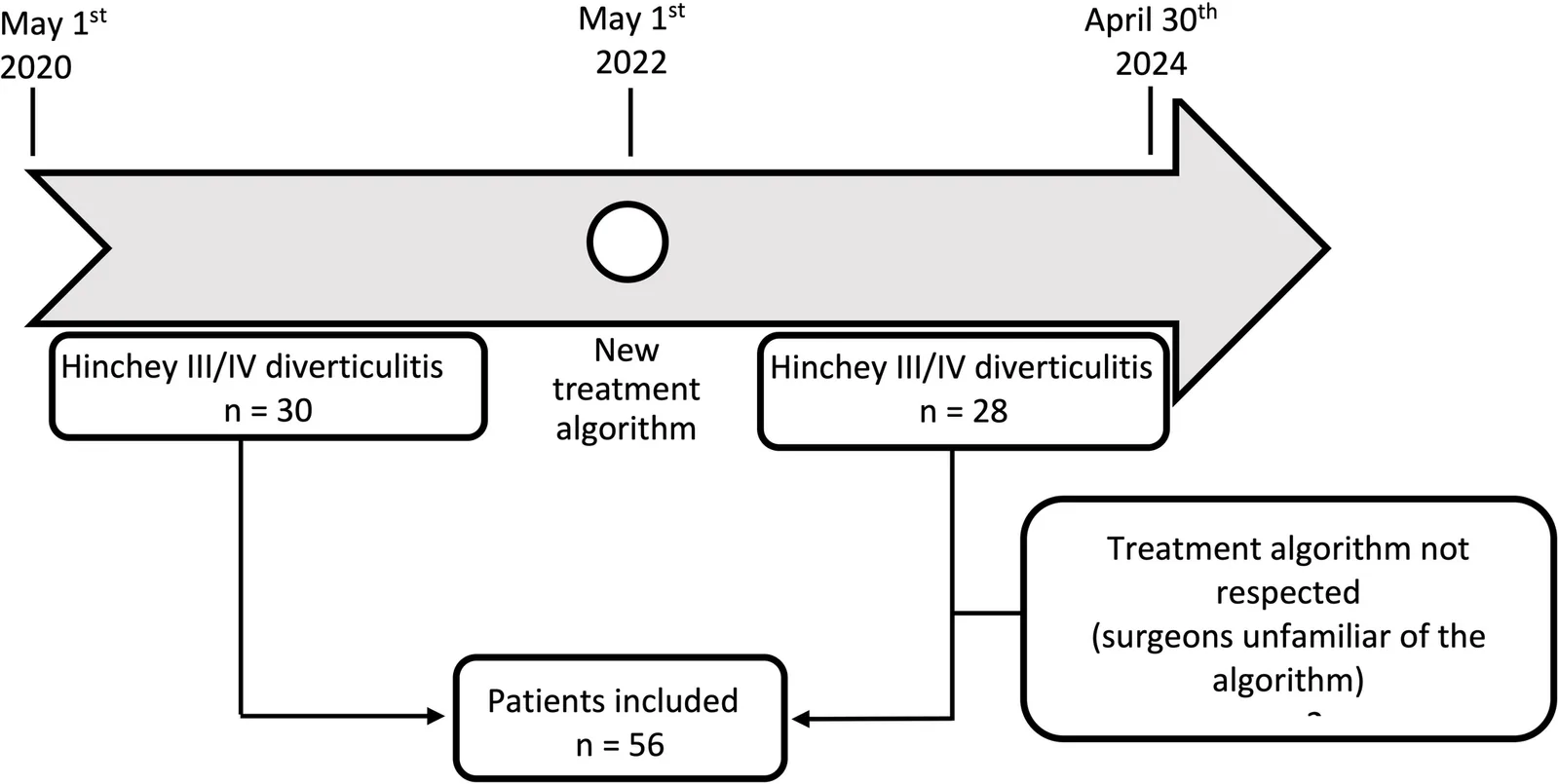
Patient inclusion flowchart. *n* = number

This before- after trial compared all patients undergoing surgery at our institution two years before and after the implementation of a new treatment algorithm based on damage control surgery. A prospective study group including patients treated from May 1 st, 2022 to April 30th, 2024 after the implementation of a new two-stage damage control treatment algorithm (Fig. 1) was compared to a retrospectively analyzed cohort (control group) including all patients treated by conventional surgical strategies from May 1 st 2020 to April 30th 2022, before implementation of the DCS treatment algorithm. Patients non treated according to the treatment algorithm were excluded from the analyses Fig. 2 presents the inclusion flowchart.

Perforated diverticulitis was defined as a free peritoneal contamination and was diagnosed based on a computed tomography (CT) scan performed at the emergency department. Cases were generally classified using the modified Hinchey classification system, with class III and IV designations used for free peritoneal perforation.

Patients in the study group were treated following the treatment algorithm illustrated in Fig. 1. Patients in the control group underwent either a NRR (Hartmann’s procedure) or a primary anastomosis with or without a diverting ileostomy depending on the surgeon’s assessment. Before the new algorithm, the in-house criteria for performing a Hartmann’s procedure were the presence of one or more of the following: (1) sepsis (sequential organ failure assessment (SOFA) score ≥ 2) (2) septic shock (vasopressors for a mean arterial pressure of ≥ 65 mmHg and lactate level > 2 mmol/l) (3) immunodeficiency (4) American Society of Anesthesiologists (ASA) score = 4, (5) severe peritoneal contamination (purulent or fecal contamination, intraoperative judgement by the operating surgeon).

We compared the data from patients treated before and after implantation of the novel treatment algorithm. The primary outcome was the presence or absence of a stoma at 12-month follow-up. Secondary outcomes were the presence or absence of stoma at discharge and 6 months, early (30 days) and late (6 months) postoperative complications, length of hospitalization, rate of restoration of bowel continuity, and post-operative complications after restoration of bowel continuity.

This study was approved by our local ethics committee.

### Clinical data

Clinical data were recorded, including age, gender, body mass index (BMI), comorbidities, immunosuppressive medications, tobacco use, ASA score, SOFA score, inflammatory markers, lactate levels at admission, need for vasopressors, and CT scan images. Procedural characteristics were also recorded, including the operation type (damage control surgery, Hartmann’s procedure, or direct anastomosis with or without diverting loop ileostomy), timing (day or night), duration of operation, and surgical approach (open or minimally invasive). Early 30-day post-operative complications were classified according to the Clavien-Dindo classification [19] and the Comprehensive Complication Index (CCI) [20]. Late post-operative complications were assessed up to the 6-month follow-up. Restoration of bowel continuity was assessed, as well as early post-operative complications after restoration of bowel continuity, using the same classification system. Costs were recorded for the initial hospitalization, rehabilitation, subsequent hospitalizations for complications, and hospitalization for the restoration of bowel continuity.

### Surgical procedures

All procedures were performed either open or minimally invasive, depending on the surgeons’ skills and patients’ characteristics. NRR (Hartmann’s procedure) comprised resection of the affected bowel segment, lavage of the peritoneal cavity, creation of an end colostomy, and abdominal wall closure. Primary anastomosis included resection of the affected bowel segment, peritoneal lavage, mobilization of the left colonic flexure, mechanical stapled intracorporeal anastomosis, and abdominal wall closure. A diverting ileostomy could be performed, according to the surgeons’ assessment.

The two-stage damage control pathway consisted of an initial damage control procedure that comprised resection of the affected bowel segment, lavage of the abdominal cavity, and either abdominal wall closure or abdominal negative pressure therapy. Next, the patients were stabilized at the intensive care or surgical unit, depending on the post-operative surveillance requirements. A second-look procedure was planned for 24–72 h later, ideally during daytime, in the presence of a senior surgeon. The second-look procedure comprised either an end colostomy or a restoration of bowel continuity (anastomosis), with or without a diverting loop ileostomy, according to the surgeon’s assessment. When necessary, mobilization of the left flexure was performed as part of the second-look procedure.

### Statistical analysis

Statistical analysis was performed using IBM SPSS Statistics Version 29.0.2.0 (IBM Corp. Released 2023; Armonk, NY, USA). The results of analyses of quantitative variables were expressed as means with standard deviations, and the results of analyses of categorical variables as numbers and frequencies. The Chi square test and Student’s t-test were used to test the independence of variables. Univariate and multivariate Cox proportional hazard regression analyses were conducted to investigate factors predictive of stoma at discharge and at the 6-month follow-up. The results were expressed as hazard ratios (HRs) with 95% confidence intervals (CIs). The level of significance was defined as *P* < 0.05.

## Results

This study included 56 patients with perforated diverticulitis (Hinchey III/IV)—including 30 in the control group (before DCS algorithm implementation) and 26 in the study group (after DCS algorithm implementation).

### Patients’ demographics and preoperative characteristics

Table 1 presents the demographic and pre-operative characteristics of both cohorts. Demographics did not significantly differ between the groups.

**Table 1:** Patients’ demographics and pre-operative data

	2020–2022 Before new algorithm *n* = 30	2022–2024 After new algorithm *n* = 26	*P* value
Age in years, mean (SD)	70.4 (12.3)	64.5 (16.0)	0.127
Sex, *n* (%)			
Male	15 (50%)	12 (46.2%)	0.774
Female	15 (50%)	14 (53.8%)	
BMI in kg/m^2^, mean (SD)	27.2 (7.1)	27.9 (5.4)	0.698
Charlton comorbidity index, mean (SD)	3.9 (1.6)	3.2 (2.6)	0.178
ASA score, *n* (%)			
I	0 (0%)	1 (3.8%)	0.160
II	6 (20%)	11 (42.3%)	
III	16 (53.3%)	8 (30.8%)	
IV	8 (26.7%)	6 (23.1%)	
V	0 (0%)	0 (0%)	
Immunosuppression,* n* (%)	6 (20%)	6 (23.1%)	0.780
Tobacco use, *n* (%)	11 (36.7%)	13 (50%)	0.315
WBC count in 10^9^/L, mean (SD)	15.4 (6.9)	15.8 (6.1)	0.798
CRP in mg/L, mean (SD)	194.8 (114.2)	219.7 (87.9)	0.371
Sofa score, *n* (%)			
< 2	8 (26.7%)	11 (42.3%)	0.218
≥ 2	22 (73.3%)	15 (57.7%)	
Hinchey classification, *n* (%)			
III	16 (53.3%)	8 (30.8%)	0.089
IV	14 (46.7%)	18 (69.2%)	
Vasopressors at induction, *n* (%)	27 (90%)	22 (84,6%)	0.543

*n*, number; SD, standard deviation; kg, kilograms; m, meter; ASA, American Society of Anesthesiologists; WBC, white blood cells; CRP, C-reactive protein

### Surgical procedures

Among the 30 patients in the control group, 29 (97%) underwent non-restorative resection (NRR), while one patient (3%) received a primary anastomosis (PRA) with a diverting ileostomy. Of the 26 patients in the study group, 4 (15.4%) underwent PRA (one with a diverting ileostomy), and 22 (84.6%) received DCS.

The reason for performing non-restorative resection in the control group was severe peritoneal contamination in 2/29 patients (7%), septic shock in 3/29 patients (10%), sepsis in 2/29 patients (7%) and immunodeficiency in 1/29 patient (3%) and more than one of the criteria (sepsis, immunosuppression, septic choc, ASA status > 4 or immunosuppression) in 19/29 patients (66%). Two out of 29 patients (7%) underwent a NNR despite the absence of one of the above-mentioned factors.

**Fig. 3:**
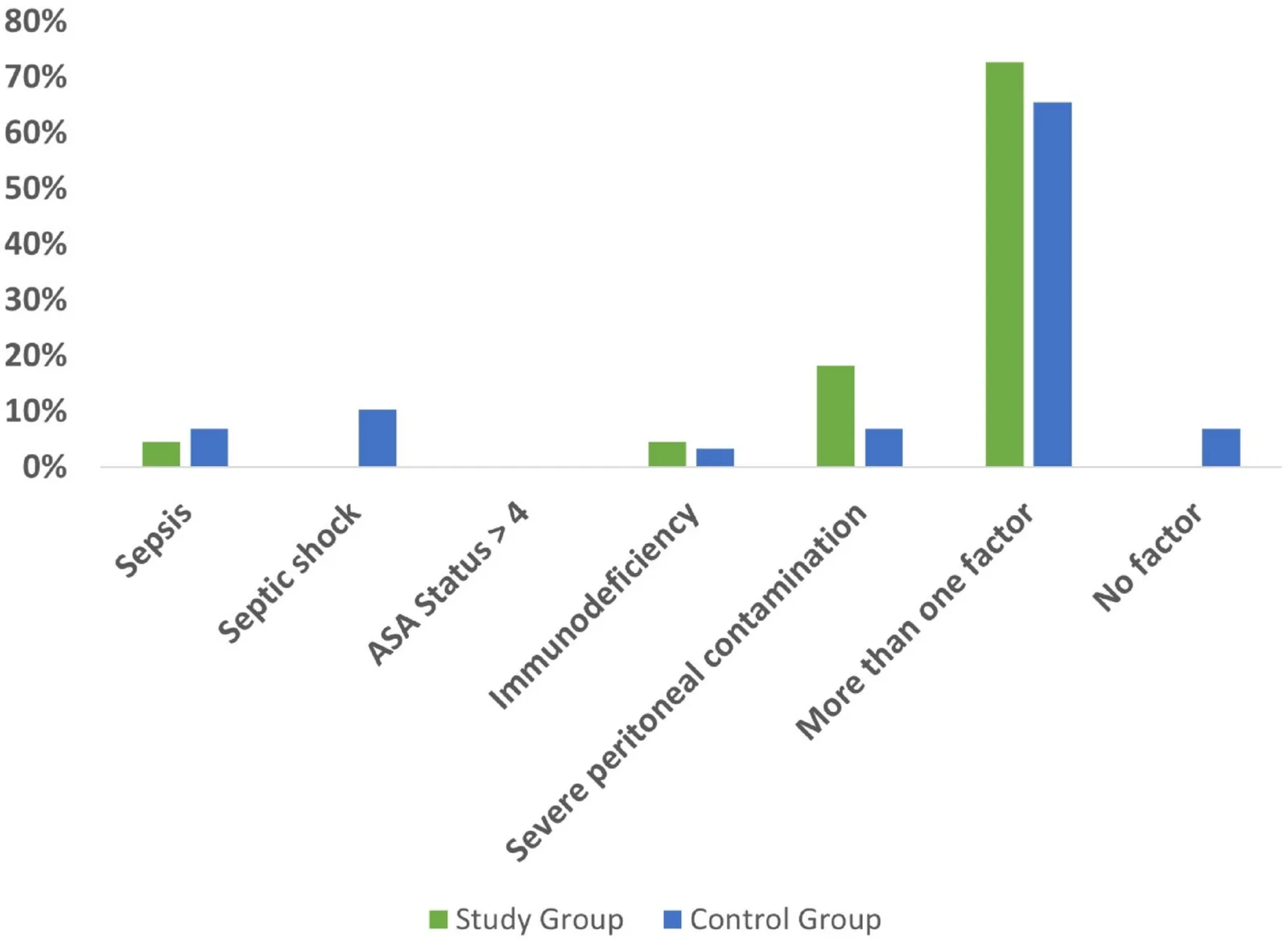
Criteria for performing non-restorative procedure in the control group and DCS in the study group. ASA= American Society of Anesthesiologists

The reasons for performing DCS in the study group were peritoneal contamination in 4/22 patients (18%), sepsis in 1/22 patients (5%), and immunodeficiency in 1/22 patients (5%). The remaining patients, 16/22 (73%) presented more than one of the criteria. No patient received DCS despite the absence of one of the above-mentioned factors. Figure 3 illustrates the reasons for a non-restorative resection or a DCS.

**Fig. 4:**
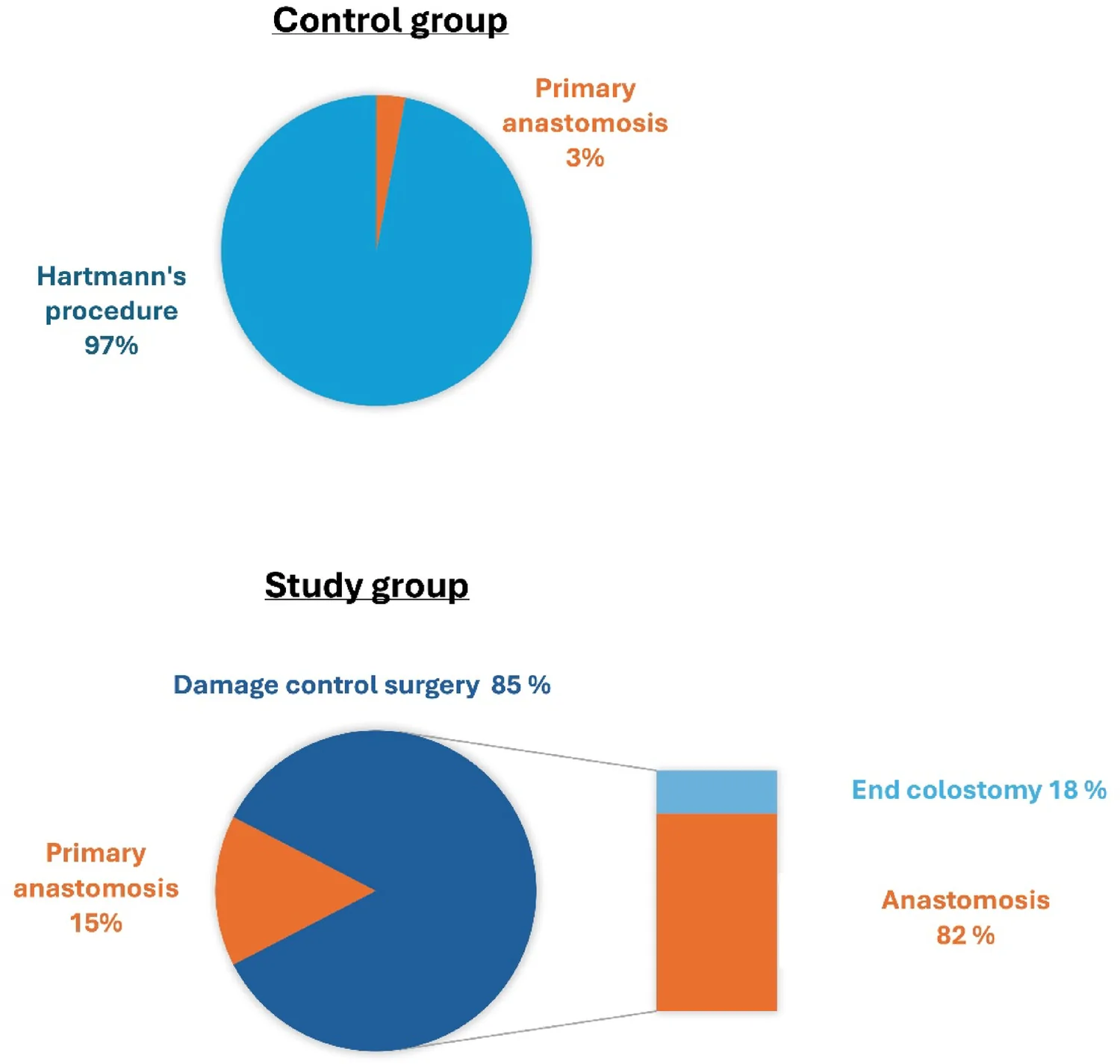
Procedures performed before and after implementation of the damage control surgery (DCS) treatment algorithm

**Fig. 5:**
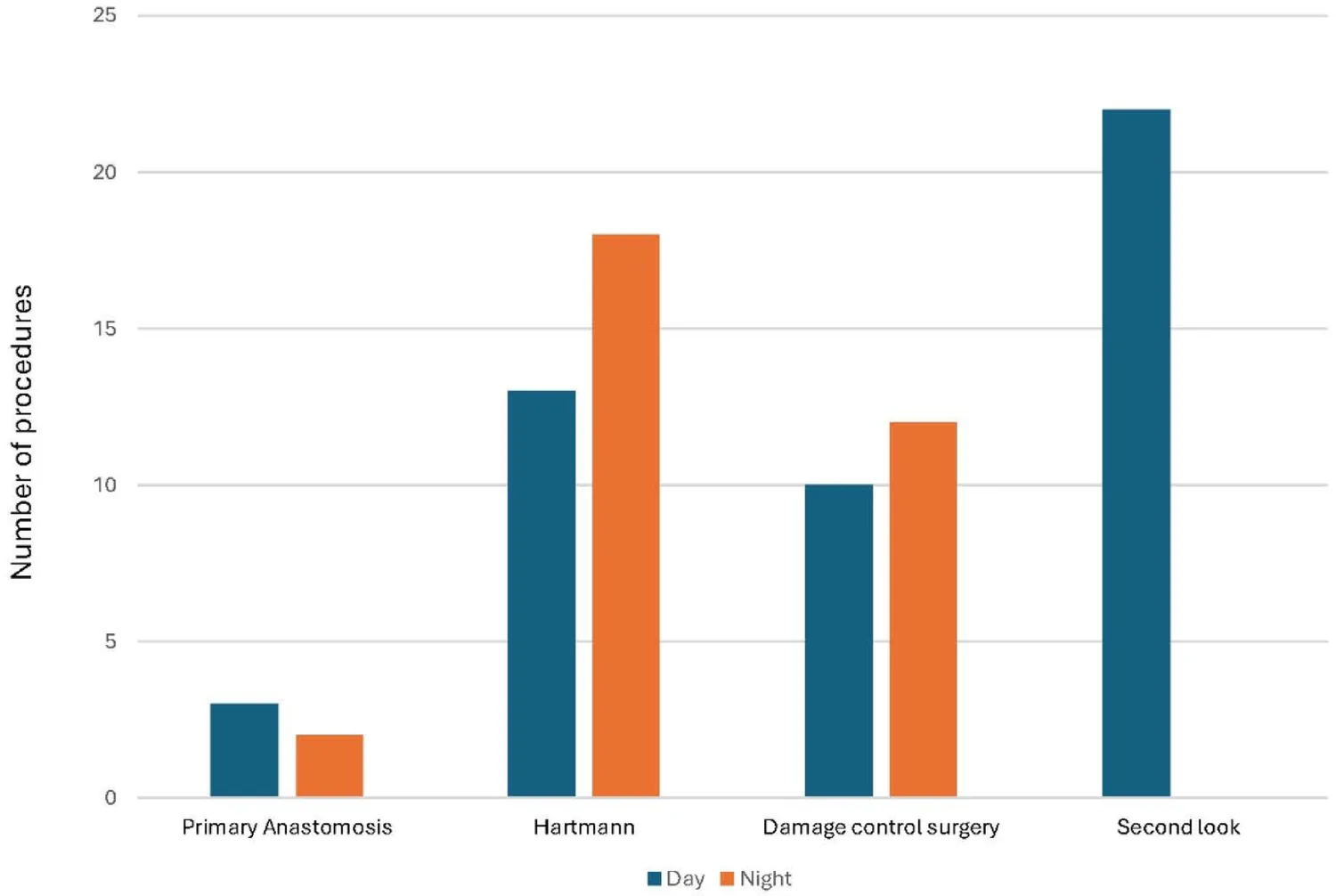
Procedures performed during the day or night

Among the 22 patients undergoing DCS, 4 (18%) required conversion to NRR during the second-look procedure, while 18 (82%) successfully underwent anastomosis (6 with a diverting ileostomy). Figure 4 presents the distribution of surgical procedures in each group. In the DSC group, all second-look procedures were performed during daytime hours (8 AM–6 PM). On the other hand, damage control procedures and NRR were more commonly performed at night (Fig. 5). The second look procedure was performed ≤ 48 h after the first look in 19/22 (86%) patients and > 48 h after the first look in 3/22 (14%).

### Perioperative outcomes

Table 2 summarizes the perioperative data and early postoperative outcomes. The operative duration for the initial procedure was significantly shorter in the DCS group (166 min) compared to the control group (220 min) (*P* = 0.011). Within the DCS group, the mean operative duration was 144 (± 63) minutes for DCS, and 190 (± 68.2) minutes for the second-look procedure. The proportion of minimally invasive procedures was higher in the study group (18%), compared to in the control group (3.3%) (*P* = 0.055). The groups did not significantly differ in terms of early post-operative complications (Clavien-Dindo classification) or length of hospitalization. The control group showed a non-significant trend towards a higher comprehensive complication index (CCI 17.1 *versus* 8.8, *P* = 0.235). The hospitalization costs were higher in the control group (56608 Confoederatio Helvetica franc (CHF) *versus* 47600 CHF), but this difference was not significantly significant (*P* = 0.429). The rate of stoma presence at discharge was significantly higher in the control group (100% *versus* 42.3%, *P* < 0.001). There was no difference in complication rates between patients having their second look procedure ≤ 48 h or > 48 h (*P* = 0.623).

**Table 2:** Peri-operative data and early post-operative outcomes

	2020–2022 Before new algorithm *n* = 30	2022–2024 After new algorithm *n* = 26	*P* value
Operative time in minutes, mean (SD)			
			**0.016**
First look	220.8 (65.4)	167.6 (93.9)	
DCS		144.1 (63.0)	
Second look		189.7 (68.2)	
Technique, *n* (%)			0.055
First look			
Open	29 (96.7%)	21 (80.8%)	
Minimally invasive	1 (3.3%)	5 (19.2%)	
Second look			
Open		19 (86.4%)	
Minimally invasive		3 (13.6%)	
Abdominal wall closure, *n* (%)			
DCS		9 (40.9%)	
Early postoperative complications, *n* (%)			
None	19 (63.3%)	19 (73.1%)	0.657
Clavien-Dindo I	3 (10%)	2 (7.7%)	
Clavien-Dindo II	2 (6.7%)	2 (7.7%)	
Clavien-Dindo IIIa	0 (0%)	0 (0%)	
Clavien-Dindo IIIb	3 (10%)	3 (11.5%)	
Clavien-Dindo IVa	0 (0%)	0 (0%)	
Clavien-Dindo IVb	2 (6.7%)	0 (0%)	
Clavien-Dindo V	1 (3.3%)	0 (0%)	
Anastomotic leakage	0 (0%)	0 (0%)	
Comprehensive complication score, mean (SD)	17.1 (30.7)	8.8 (18.4)	0.235
Length of stay in days, mean (SD)	18.8 (16.0)	14.8 (9.1)	0.279
Hospitalization cost in CHF, mean (SD)	56,608 (51,423)	47,600 (18,405)	0.429
Stoma at discharge, *n* (%)	30 (100%)	11 (42.3%)	** < 0.001**
End colostomy	29 (96.7%)	4 (15.4%)	
Protective ileostomy	1 (3.3%)	7 (26.9%)	

*n*, number; SD, standard deviation; DCS, damage control surgery; CHF, confoederatio helvetica francs

### Follow-up outcomes

Table 3 presents the follow-up data. Ileostomy closure was performed in all patients who had an ileostomy in both groups. Restoration of bowel continuity after NRR was achieved in 66% of the control group, and 50% of the study group (*P* = 0.361). The groups did not significantly differ in early postoperative complications following bowel continuity restoration, expressed by the comprehensive complication index. However, the control group exhibited significantly higher rates of late post-operative complications (55.6% *versus* 26.9%, *P* = 0.034), which were mostly wound-related (40%), and a significantly higher rate of stoma presence at 6 months (43.3% *versus* 7.8%, *P* = 0.003) and 12 months (36.7% *versus* 7.8%, *P* = 0.01). The costs of hospitalization for restoration of bowel continuity were higher in the control group (36876 CHF *versus* 29467 CHF); however, this difference was not statistically significant (*P* = 0.537).

**Table 3:** Long-term outcomes

	2020–2022 Before new algorithm *n* = 30	2022–2024 After new algorithm *n* = 26	*P* value
Ileostomy closure, *n* (%)	1/1 (100%)	7/7 (100%)	0.999
Restoration of bowel continuity, *n* (%)	19/29 (65.5%)	2/4 (50%)	0.361
Comprehensive complication index following ileostomy closure/restoration of bowel continuity, 30 days, mean (SD)	10.2 (18.10)	4.7 (11.3)	0.406
Hospitalization cost (restoration) in CHF, mean (SD)	36,876 (29,522)	29,467 (18,394)	0.537
Late postoperative complications, *n* (%)			
None	12 (44.4%)	19 (73.1%)	**0.034**
Incisional hernia	5 (16.6%)	4 (15.4%)	
Parastomal hernia	1 (3.3%)	0 (0%)	
Stoma prolapse	1 (3.3%)	1 (3.8%)	
Wound complications	6 (20%)	0 (0%)	
Other	1 (3.3%)	1 (3.8%)	
Stoma at 6-month follow-up, *n* (%)	13 (43.3%)	2 (7.8%)	**0.003**
End colostomy	13 (43.3%)	2 (7.8%)	
Protective ileostomy	0 (0%)	0 (0%)	
Stoma at 12-month follow-up, *n* (%)	11 (36.7%)	2 (7.8%)	**0.010**
End colostomy	11 (36.7%)	2 (7.8%)	
Protective ileostomy	0 (0%)	0 (0%)	

*n*, number; CHF, confoederatio helvetica francs; SD, standard deviation

#### Predictors of stoma at discharge and 6 months

Univariate regression analysis identified comorbidities, lactate levels, and group allocation as predictors of stoma presence at discharge; and comorbidities and group allocation as predictors of stoma presence at 12 months. In multivariate regression analysis, only prior treatment strategy (conventional *versus* DCS) remained a significant predictor of stoma presence at discharge (HR 13.1, 95% CI 0.9–66.2, *P* = 0.05) and at 12 months (HR 10.7, 95% CI 1.6–69.3, *P* = 0.013) (Tables 4 and 5).

**Table 4:** Univariate and multivariate analysis of factors associated with stoma presence at discharge, with hazard ratios and 95% confidence intervals

	Univariate	*P* value	Multivariate	*P* value
Study/control group Ref. study group	39.56 (4.65–3936.19)	**0. 001**	13.05 (0.93–66.24)	**0.05**
Age	1.02 (0.98–1.06)	0.277	1.00 (0.92–1.09)	0.924
Gender Ref. male	0.78 (0.24–2.49)	0.673	0.66 (0.08–5.62)	0.702
Comorbidities	1.65 (1.14–2.39)	**0.009**	18.72 (0.61–55.05)	0.09
Immunosuppression Ref. none	5.69 (0.67–48.36)	0.111	1.89 (0.13–27.1)	0.132
Hinchey classification Ref. Hinchey III	0.73 (0.22–2.41)	0.609	3.17 (0.27–36.3)	0.353
Lactate level	**4.33 (1.22–15.41)**	**0.023**	0.34 (0.09–1.27)	0.108
Vasopressors Ref. none	**4.11 (0.80–21.01)**	0.090	0.17 (0.00–83.05)	0.572

Ref, reference

**Table 5:** Univariate and multivariate analysis of factors associated with stoma presence at 12 months, with hazard ratios and 95% confidence intervals Ref, reference

	Univariate	*P* value	Multivariate	*P* value
Study/control group Ref. study group	9.17 (1.83–46.01)	**0. 007**	10.65 (1.64–69.29)	**0.013**
Age	0.43 (1.26–1.49)	0.183	0.94 (0.86–1.02)	0.145
Gender Ref. male	0.17 (0.01–2.20)	0.174	2.15 (0.41–11.39)	0.174
Comorbidities	0.52 (0.34–0.79)	**0.002**	7.82 (0.34–179.39)	0.198
ImmunosuppressionRef. none	1.50 (0.38–5.97)	0.565	1.31 (0.24–7.09)	0.751
Postoperative complications Ref. none	0.98 (0.96–1.00)	0.053	0.98 (0.95–1.01)	0.163

Ref, reference

### Subgroup analysis

We performed a subgroup analysis excluding patients who underwent primary anastomosis as a first look procedure in the control and study group to compare only patients undergoing Hartmann’s procedure with patients undergoing DCS (Tables 1, 2, 3, 4 and 5*). In multivariate regression analysis, only prior treatment strategy (Hartmann’s *versus* DCS) remained a significant predictor of stoma presence at discharge (HR 10.3, 95% CI 0.9–125.3, *P* = 0.05) and at 12 months (HR 12.4, 95% CI 1.8–86.3, *P* = 0.011).

## Discussion

In this study, we evaluated the efficacy of damage-control surgery (DCS) for perforated diverticulitis, a strategy not yet widely adopted in current guidelines. European and American guidelines for perforated diverticulitis treatment recommend that resection with primary anastomosis can be performed in hemodynamically stable immunocompetent patients [1–4]. This recommendation is largely based on prospective trials that have compared PRA (protected by diverting ileostomy) to NRR. Such trials have demonstrated that the procedures are associated with similar morbidity and mortality rates, but NRR carries a higher rate of permanent stoma, longer time to stoma reversal, and increased post-reversal complications [6, 9, 21–23]. Despite these findings, NRR remains the most common approach in emergency settings [12, 24, 25], largely due to concerns about the technical challenges of PRA (particularly in cases of purulent or fecal peritoneal contamination), and the potential for life-threatening anastomotic leakage, especially when performed at night and/or by less experienced surgeons. However, it has been established that stomas have negative impact on patients’ quality of life [26, 27], underscoring the need for strategies to minimize stoma creation.

In recent years, DCS has emerged as a promising alternative for managing intra-abdominal sepsis [16], particularly perforated diverticulitis [10, 13, 15, 17, 18, 28]. Our present results align with this trend, showing that bowel continuity without stoma was achieved during the index hospitalization in 58% of patients treated using the DCS algorithm, compared to 0% in the control group (NRR/PRA). This significant improvement was achieved without increases of early postoperative complications, duration of hospital stay, or hospitalization costs. Moreover, the DCS strategy was associated with markedly lower rates of stoma presence at 6 and 12 months (*P* < 0.05). The staged approach of DCS offers the advantage that the initial phase allows for stabilization of critically ill patients, while the second-look procedure provides the opportunity for anastomosis under optimal conditions. Therefore, the DCS strategy reduced NRR procedures in initially unstable patients, who were stabilized by the time of the second-look procedure, as well as reduced NRR in stable patients where PRA would not be performed due to apprehension regarding complications, e.g., in cases of fecal peritonitis or patients with multiple comorbidities.

Partly, the higher rate of stoma free discharge and absence of stoma at 12 months can be explained by the higher rate of primary anastomosis in the study group (15%) compared to the control group (3%). Indeed, 2 patients (7%) in the control group underwent a NRR despite the absence of criteria contra-indicating a primary anastomosis. This higher rate of primary anastomosis in the study group could reflect the influence of an existing algorithm on intraoperative decision making or an evolving paradigm shift in surgical practice. Our subgroup analysis after exclusion of patients treated by primary anastomosis at the first look showed, however, a higher rate of stoma free discharge and absence of stoma at 12 months in patients treated by DCS compared to those treated by NRR in the control group.

While DCS offers the potential to reduce stoma rates and improve patient outcomes, this study also highlighted potential drawbacks. The staged nature of DCS inevitably increases the risk of overtreatment. Patients who might otherwise be successfully treated by PRA could undergo an unnecessary second operation. To mitigate this, we implemented a treatment algorithm based on the criteria proposed by Faes et al. [16], which successfully stratified patients for appropriate treatment (85% DCS, 15% PRA in the study group). Treating all patients with perforated diverticulitis by DCS would have overtreated some of the patients.

There is also a risk of overtreatment among patients in whom anastomosis might never be performed—for instance, due to fecal incontinence or immunosuppression. The application of the treatment algorithm should be refined to carefully select patients who are appropriate candidates for DCS.

Another limitation for the damage control approach can be the need for additional emergency theatre resources for the second look procedure with consequential organization issues and financial overload.

The present study was limited by its retrospective nature, potential selection bias, relatively small sample size and the lack of long-term outcomes (> 3 years). The retrospective design of the control group introduced inherent limitations regarding data completeness and the possibility of selection bias. The small cohort size restricts the statistical power of our analysis and may influence the significance of certain observations. Future prospective randomized controlled trials are needed to definitively confirm the efficacy and safety of DCS compared to conventional approaches for managing perforated diverticulitis. Further investigation is warranted to optimize patient selection, and to refine the algorithm to further reduce the need for stomas.

In conclusion, while the surgical management of perforated diverticulitis remains a subject of debate, our present findings support that DCS may be a valuable alternative for appropriately selected patients, potentially offering significantly increased stoma-free discharge rates, and reduced definitive stoma rates, without increasing morbidity or mortality.
